# Influence of Bone Definition and Finite Element Parameters in Bone and Dental Implants Stress: A Literature Review

**DOI:** 10.3390/biology9080224

**Published:** 2020-08-14

**Authors:** María Prados-Privado, Carlos Martínez-Martínez, Sergio A. Gehrke, Juan Carlos Prados-Frutos

**Affiliations:** 1 Asisa Dental, Research Department, C/José Abascal, 32, 28003 Madrid, Spain; carlos.martinez@asisa.es; 2 Department of Signal Theory and Communications, Higher Polytechnic School, Universidad de Alcalá de Henares, Ctra. Madrid-Barcelona, Km. 33,600, Alcalá de Henares, 28805 Madrid, Spain; 3 Department Continuum Mechanics and Structural Analysis, Higher Polytechnic School, Carlos III University, Avenida de la Universidad 30, Leganés, 28911 Madrid, Spain; 4 IDIBO GROUP (Group of High-Performance Research, Development and Innovation in Dental Biomaterials of Rey Juan Carlos University), Avenida de Atenas s/n, Alcorcón, 28922 Madrid, Spain; 5 Department of Research, Biotecnos, Cuareim 1483, Montevideo CP 11100, Uruguay; sergio.gehrke@hotmail.com; 6 Department of Medicine Specialties and Public Health, Faculty of Health Sciences, Rey Juan Carlos University, Avenida de Atenas s/n, Alcorcón, 28922 Madrid, Spain; juancarlos.prados@urjc.es

**Keywords:** finite element, dental implants, bone, stress, review

## Abstract

Bone plays an important role in dental implant treatment success. The goal of this literature review is to analyze the influence of bone definition and finite element parameters on stress in dental implants and bone in numerical studies. A search was conducted of Pubmed, Science Direct and LILACS, and two independent reviewers performed the data extraction. The quality of the selected studies was assessed using the Cochrane Handbook tool for clinical trials. Seventeen studies were included. Titanium was the most commonly-used material in dental implants. The magnitude of the applied loads varied from 15 to 300 N with a mean of 182 N. Complete osseointegration was the most common boundary condition. Evidence from this review suggests that bone is commonly defined as an isotropic material, despite being an anisotropic tissue, and that it is analyzed as a ductile material, instead of as a fragile material. In addition, and in view of the data analyzed in this review, it can be concluded that there is no standardization for conducting finite element studies in the field of dentistry. Convergence criteria are only detailed in two of the studies included in this review, although they are a key factor in obtaining accurate results in numerical studies. It is therefore necessary to implement a methodology that indicates which parameters a numerical simulation must include, as well as how the results should be analyzed.

## 1. Introduction 

Dental implants are a common practice in dentistry, and have the goal of transferring bite loads to the bone and the surrounding tissues [1]. Therefore, implants must be designed to distribute loads to the surrounding tissue in an optimized way.

Two types of bone can be distinguished, both in the mandible and maxilla: cortical and cancellous bone. Both cortical and cancellous are anisotropic materials, and the difference between them is that trabecular is a compact bone and cancellous is a highly porous mineralized tissue enclosed in cortical bone [2]. 

Load transfer from dental implants to bone is influenced by several factors such as the material, the magnitude and angulation of the load, the design of dental implant (mainly, length and diameter) or the quantity and quality of the surrounding bone [3]. 

There are several tools to analyze the biomechanical behavior of dental implants and bone, including in vitro techniques and numerical simulations. In vitro studies include photoelastic stress analysis or mechanical fracture tests, e.g., of static and dynamic performance, for which there are international standards [4]. 

The most common numerical simulation is the finite element method, a numerical technique to simulate different conditions which obtains results with good accuracy but which strongly depends on the quality of the mesh, the precision of data and boundary and loading conditions [5]. Numerical studies are usually carried out in programs such as ANSYS or Abaqus, which are programs where geometry, material and load data are introduced, while discretization is carried out through meshing; then, it is run and the results of the required stress or strain are obtained. 

The goal of this review is to analyze the influence of bone definition and finite element parameters on stress in dental implants and bone in numerical studies. Relevant data about dental implants, such as material, connection, geometry and manufacturer, were analyzed. The most important data about finite element parameters, i.e., mesh characteristics, bone properties, loading and boundary conditions and convergence criteria, were detailed and reviewed. Finally, stress values in bone and dental implants, as well as the calculus criterion, were analyzed. 

## 2. Materials and Methods 

### 2.1. Review Questions

The research questions were elaborated considering each of the components of the PICO(S) [6] strategy research questions, which are explained as follows: (P) bone definition and finite element parameters; (I) loading and boundary conditions; (C) studies with implants where bone and dental implant materials are defined; (O) evaluation of the measured stress and its values in bone and dental implants; (S) finite element study.

### 2.2. Search Strategy

An electronic search was performed of the MEDLINE/PubMed, Science Direct and LILACS databases prior to 1 July 2020. The search strategy used is detailed in Table 1.

### 2.3. Study Selection

M.P.-P. and J.C.P.-F. performed bibliography searches and selected articles that fulfilled the inclusion criteria. Both authors collected all the data from the selected articles in duplicate and independently of each other. The references of the articles included in this study were manually reviewed.

### 2.4. Inclusion and Exclusion Criteria

Inclusion criteria were full manuscripts including conference proceedings that report the use of finite element analysis and bone properties to determine the stress on bone and dental implants under certain conditions. There were no restrictions on the language or date of publication. Exclusion criteria were reviews, no dental application, no stress measures on bone and/or dental implant, zygomatic implants, short or mini implants and protheses. 

### 2.5. Study Quality Assessment

The risk of bias from finite element studies was evaluated by two of the authors (C.M.-M. and S.A.G.). To this end, the guidelines presented in the Cochrane Handbook [7] were followed, which incorporates seven domains: random sequence generation (selection bias); allocation concealment (selection bias); masking of participants and personnel (performance bias); masking of outcome assessment (detection bias); incomplete outcome data (attrition bias); selective reporting (reporting bias); and other biases.

The studies were classified into the following categories: low risk of bias—low risk of bias for all key domains; unclear risk of bias—unclear risk of bias for one or more key domains; high risk of bias—high risk of bias for one or more key domains.

### 2.6. Statistical Analysis

The mean, standard deviation (SD) and percentage were calculated for several variables. Statistical calculations were performed with IBM SPSS Statistics (SAS Institute Inc., Cary, NC, USA).

## 3. Results

### 3.1. Study Selection

**Figure 1:**
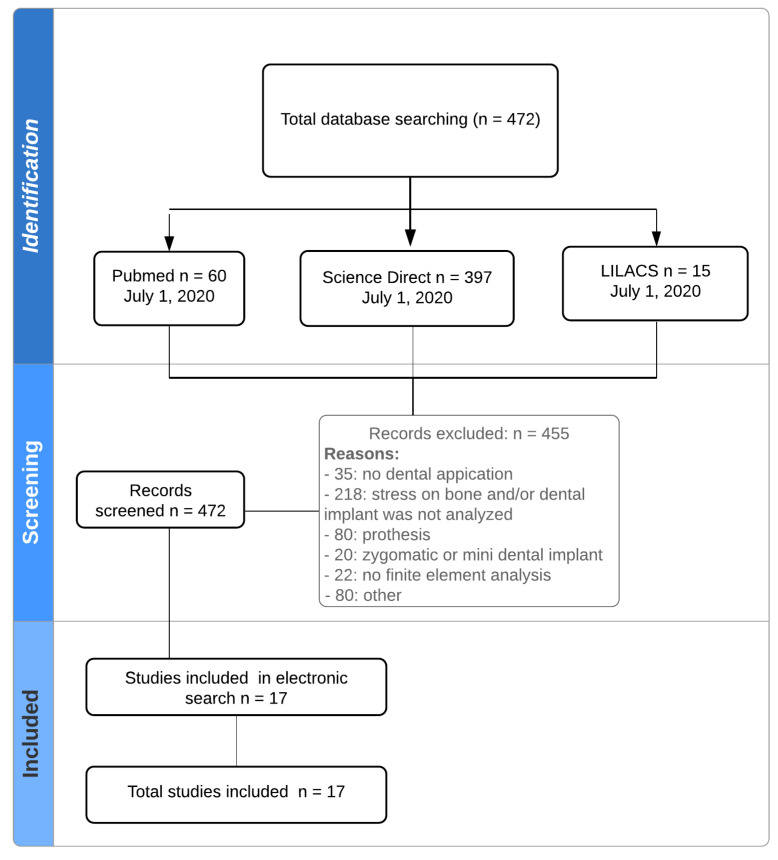
Flowchart.

Figure 1 presents a flowchart of the study selection. All electronic search strategies provided 472 potential manuscripts. A total of 455 studies were excluded because they did not meet the inclusion criteria. Additionally, a manual search was carried out to analyze the references cited in 17 of the articles that were included in this work. No more articles were incorporated from the manual search. In the end, a total of 17 studies were analyzed. 

### 3.2. Relevant Data of Included Studies Regarding Dental Implants

Table 2 details the main characteristic of the dental implants analyzed in the included manuscripts.

In view of Table 2, most of the manuscripts included in this study (n = 17) employed dental implants made of titanium; only one of them analyzed zirconia dental implants. The Young’s modulus ranged between 104 and 116 GPa (mean ± SD of 115.72 ± 23.62 GPa), with 110 GPa being the widely used value (77.8% of the studies). Regarding the diameter and length of dental implants, there is a wide variety of options, so, in this sense, the geometric characteristics of the implants are not homogenized. The diameters of the implants in the included studies had a mean ± SD of 4.09 ± 0.27 mm (min–max, 3.7–4.8). The external (n = 6) connection was most widely employed in the studies included in this review, followed by the internal (n = 3) connection. However, n = 12 studies did not detail the type of dental implant connection employed in their analysis. Finally, Biomed 3i was the most widely used manufacturer (n = 3), followed by ITI (n = 2) and Ankylos, Anthogyr, Straumann, Nobel Biocare and Warantec (n = 1, each one). n = 10 studies did not indicate the manufacturer of the implant used in their studies.

### 3.3. Relevant Data of Finite Element Parameters

Table 3 details the most important parameters to describe the finite element method in each study included in this review. In view of Table 3, n = 15 studies analyzed bone as an isotropic material, n = 4 analyzed it as an anisotropic and n = 1 study characterized it as a transversely isotropic material. Regarding the software employed, n = 9 studies used ANSYS (in their different versions), n = 2 employed Pro/Engineer and n = 1 each employed AMMEDYSA, ABAQUS and COMSOL. However, n = 4 studies did not specify the software employed to perform the numerical analysis.

The number of nodes ranged from 14,805 to 1,368,886 (mean ± SD of 261,013.09 ± 443,988.78), and the number of elements between 42,740 and 704,068 (mean ± SD of 149,512.32 ± 178,653.46), although n = 5 studies did not detail the number of nodes and elements that conformed their mesh. Regarding mesh characterization, n = 6 employed tetrahedral elements, n = 4 employed a quadratic element and n = 8 studies did not provide information about the type of element employed in the mesh. Finally, only n = 2 studies detailed the convergence criterion to validate the results obtained by the analysis.

Regarding boundary conditions, n = 7 studies detailed a 100% osseointegration, n = 14 employed fully constrained restrictions in any of the axes and n = 4 studies did not explain the boundary conditions employed.

Studies included in this review employed horizontal, vertical and oblique loads. n = 4 studies used horizontal loads, n = 6 employed vertical loads and n = 6 oblique loads. The magnitude of the loads ranged from 40 to 800 N (mean ± SD of 182.41 ± 93.35 N).

**Figure 2:**
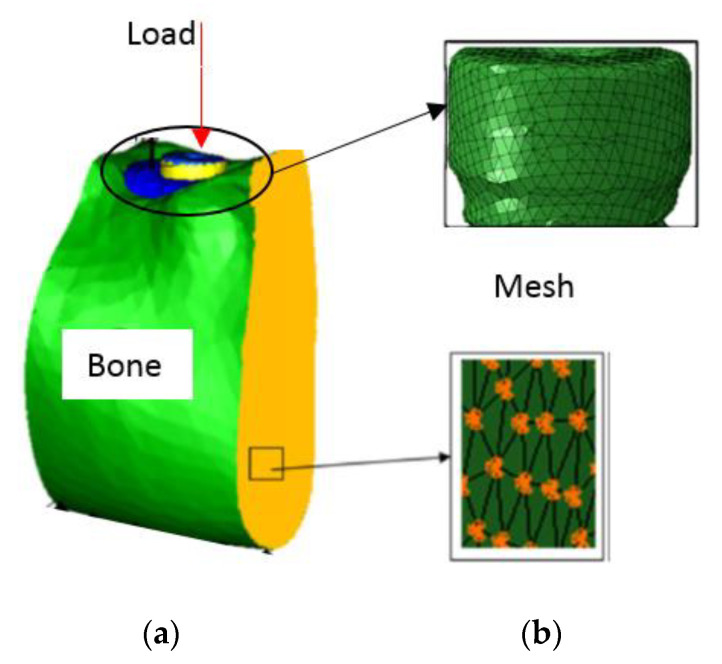
Three-dimensional model. (**a**) Bone and dental implant assembly; (**b**) Mesh.

Figure 2 provides an example of a 3D model to simulate the finite element analysis.

Of the included studies, 66.7% analyzed bone as an isotropic material, followed by 14.3%, which analyzed it as an anisotropy material. Only one study compared the differences of defining bone as an isotropic or anisotropic material. The cortical bone Young’s modulus ranged from 12.6 to 26.6 GPa (mean ± SD of 15.46 ± 3.95 GPa) and that of cancellous bone ranged from 0.021 to 1.5 GPa (mean ± SD of 1.09 ± 0.44 GPa). Poisson’s ratio ranged from 0.18 to 0.39 (mean ± SD 0.29 ± 0.043) in cortical bone and from 0.01 to 0.322 (mean ± SD 0.21 ± 0.12) in cancellous bone.

### 3.4. Stress on Bone and Dental Implant

Table 4 details the stress values on dental implants and on bone reported in the studies included in this review. Most of the studies employed von Mises stress criteria for both dental implants and bone. n = 3 studies employed maximum principal stress to calculate the stress on bone. The mean stress in dental implants in the included studies was 285.8 MPa (SD of 309.75 MPa). The mean stress in bone was 216.6 MPa (SD of 359.39 MPa). The study that analyzed isotropic and anisotropic bone reported an increase of 78% in dental implant stress and an increase of 75% in bone stress.

### 3.5. Study Quality Assessment

Evaluation of selection bias: None studies indicated whether there was concealment of this allocation.

Evaluation of performance bias: In all the studies analyzed, there was no blinding of staff or assessors.

Assessment of detection bias: The results were not blinded in any of the studies.

Evaluation of attrition bias: Not all the studies reported complete results; only seven detailed all the results analyzed in the present review [13,16,17,18,19,20,22].

Evaluation of notification bias: Not all the studies provided detailed information about the parameters employed in the finite element analysis. Cos Juez et al. [22] provided all the information.

**Figure 3:**
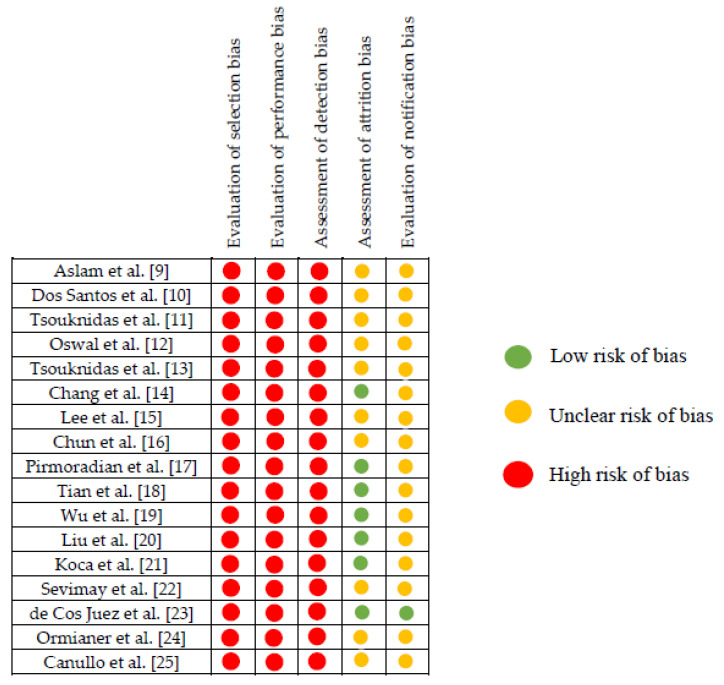
Assessment of risk of bias of included studies.

Figure 3 shows a detailed description of the risk assessment of bias in the included studies.

## 4. Discussion

The use of dental implants and implant-supported prostheses is a common practice in oral rehabilitation of patients with missing teeth [25]. Due to the success of implant treatments being influenced, among other factors, by the quality of bone, the goal of this review is to analyze the influence of bone definition on stress in dental implants and bone in finite element studies in the dentistry field.

Three hundred and ninety-seven papers were found in Science Direct, 60 in Pubmed and 15 in LILACS. However, after screening, most of the studies included in this review were taken from Pubmed, which is the major source for medical and dental research.

Finite elements have become an excellent tool in the field of medicine in general, and in dentistry in particular. The results provided by numerical simulations depend heavily on the finite elements parameters, that is, mesh definitions, mesh elements, material properties and boundary and loading conditions.

Finite element analysis subdivides the domain into smaller parts, which is a discretization of the domain. This discretization is implemented by the mesh. The objective of the mesh is to obtain results regarding elements’ vertices; therefore, the finer and better it is defined, the better the results that will be obtained. The results obtained by finite element analyses are very dependent on the mesh, which depends on the shape of the element and the number of elements. To know when the results can be considered valid, a convergence criterion must be used, which indicates when the mesh is good enough. Two of the studies employed a convergence criterion to analyze the effect of the element size on the results. These two studies cannot be compared, because different conditions were employed.

Another important factor in mesh definition is the element type. One study detailed the use of a tetrahedral element, i.e., an element with three degrees of freedom at each node. Two studies used quadratic tetrahedral elements i.e., elements with 10 nodes with three degrees of freedom at each node. Three studies employed a SOLID187 element, i.e., a 10-node element with quadratic displacement behavior employed to model irregular geometries [26].

Titanium is commonly employed to manufacture dental implants due to its superior properties, such as high strength and stiffness, good corrosion and oxidation resistance, and good biocompatibility [27,28,29]. However, a new material is increasingly being used as an alternative to conventional titanium in dental implants, i.e., zirconia. Some studies analyzed the advantages and inconveniences of using zirconia vs. titanium in dental implants [30,31]. The mechanical and physical properties of zirconia depend of its composition, the nature of the crystals, the percentage of stabilizing metal oxide, among other factors [30]. The main advantage of zirconia over titanium is aesthetic nature, due to the silver color of titanium. One of the disadvantages reported is the early fracture of one-piece zirconia dental implants, which is a critical factor in clinical practice, especially in posterior regions [30]. Only one of the studies included in this review employed zirconia dental implants. In view of the results of the zirconia study, the stress obtained both in the dental implant and bone is lower than the average of the studies whose implants were made of titanium.

Titanium–Niobium (Ti-Nb) binary alloy is a promising new material for biomedical applications; it has been proposed as an alternative to Ti6Al4V in dental implants [32], due to its osseointegration, high strength, superior biocompatibility, and nontoxic properties [29,33]. Compared with Ti-base alloy, Ti-Nb possesses a low elastic modulus (E) because of the addition of Nb. Some in vitro and in vivo studies have concluded that the mechanical properties of Ti-Nb alloys depend on the Nb content [34,35]. However, more studies are needed to explore and compare the properties of Ti-Nb to those of Ti6Al4V or commercial pure titanium (cp-Ti) regarding cytocompatibility, corrosion resistance, and altering the surface composition of alloys after prolonged exposure to physiological fluids [36].

The material properties of titanium were defined with Young’s modulus, which ranged from 110 to 116 GPa, and Poisson’s ratio, which ranged from 0.33 to 0.37. Several studies concluded, and it is well known, that bone is an anisotropic tissue [2]. This means that bone properties are different in different directions. Four of the included works studied bone as anisotropic material; however, most of the studies available in the scientific literature analyzed bone as isotropic. The only study included in this review that compared stresses when the material was isotropic or anisotropic under the same conditions concludef that, in both bone and implant, stresses were higher in the case of anisotropy.

Loading conditions also varied between the included manuscripts. Most of the included studies applied an oblique load, detailing the magnitude of the vertical and horizontal load or the magnitude of the load and the degree. The applied loads varied from 15 to 300 N, with a mean of 182 N.

Some diseases, such as bruxism or orthodontic defects, can be studied with loading conditions. Bruxism has been, and continues to be, considered a potential risk factor of dental implant treatment and its success. The main characteristics of bruxism are clenching or grinding of the teeth and bracing or thrusting of the mandible [37,38]. Bruxism is analyzed with bigger load magnitude than the common bite load. Lan et al. studied bruxism in a dental implant under 800 N of loading [39]. It was found that bruxism causes overload, which may also cause the reabsorption of bone, leading to biological complications. None of the studies included in the present review analyzed bruxism.

Orthodontic tooth movement can be also studied with finite elements. When a mechanical force acts on a target tooth, it creates stresses, resulting in inflammatory mediators in the periodontal ligament [40]. In this case, the load magnitude can be close to 0.46 N [41], which is a much smaller value than those applied in the conventional implants discussed in this review.

The boundary conditions also differed among the studies. Seven studies simulated a complete (100%) osseointegration, and one simulated a good osseointegration, but without specifying how it had been detailed. All studies included in this review constrained a surface, and four did not simulate complete osseointegration, but instead, applied contact conditions to simulate a more real bone condition around the implant.

Also, due to the fact that bone is a brittle material, the appropriate way to define it is via maximum principal stress [42]; however, only three of the included studies employed maximum principal stress. Due to the heterogeneity of the conditions, it was difficult to find differences between the effect of studying bone with von Mises and with maximum principal stress.

The most common diameters in dental implants have historically ranged from 3.75 to 4.1 mm, i.e., standard-diameter implants, which provide excellent long-term properties. However, some studies have reported that bone stress increases with dental implant diameter, thereby jeopardizing biomechanical behavior [43]. Among the exclusion criteria were mini implants (i.e., with diameters less than 3 mm), so all diameters of the included studies were within the conventional diameter range. However, the lengths varied between 8.5 and 15 mm. Five of the included studies did not provide any information about the geometric characteristics of the implant used. Two studies analyzed an implant with the same geometric characteristics, very similar material conditions and the same applied load; nevertheless, they reported significantly different stresses, i.e., multiplied by five in the implant and double in the bone stress values. These differences may be due to the mesh, since one of the studies did not detail the values of the mesh used, confirming the importance of correctly defining the mesh parameters.

Patient health or prothesis, among others, are factors related to implant success and bone stress. Some studies have concluded that prothesis failure is influenced by the cantilever and parafunctional habits (e.g., bruxism) [44]. Smoking can also increase the probability of dental implant failure [45].

None of the manuscripts included in this review included a long-term analysis of implants. Dental implants are subjected to cyclical loads from chewing, which leads to the appearance of a fatigue phenomenon which must be studied in depth when analyzing the success of these treatments. A good, long-term analysis is crucial in patients with diseases such as bruxism.

A qualitative analysis of the included manuscripts was done by assessing the risk of biases. The goal of this analysis was to check that all data had been managed in a controlled manner [7]. All manuscripts included in the present review had a high risk of bias. The guidelines described in the Cochrane Handbook were employed to assess the risk of bias, noting that in the first three domains, all manuscripts had high risk of bias, due to the lack of information related to allocation masking and the blinding of staff and data assessors.

The high risk of bias, together with the heterogeneity of the available results and the heterogeneity of the definition of finite elements, such as mesh and type of material, led us to interpret the results with caution. It is important to describe the external validity of the study, because this factor helps us to know if the results can be applied to other individuals or scenarios. Most of the finite element studies available in the literature analyzed a section of bone with an implant. However, other factors, such as the number of patients, their position or their oral health, among others, were not analyzed.

## 5. Conclusions

In view of the data analyzed in this review, it can be concluded that there is no standardization for conducting finite element studies in the field of dentistry. It is therefore necessary to apply a methodology that indicates which parameters a numerical simulation must include, as well as how the results should be analyzed.

The definition of bone as an isotropic material is extended in finite element studies in the field of dentistry, without being able to make any conclusive statement due to the variety of conditions used in the studies analyzed.

The limitations found and detailed in this review did not allow us to report consistent data. The present review presents, in a concise way, how finite elements are applied in the field of odontology, and helps researchers to understand the state of the field of study at present.

## Figures and Tables

**Table 1:** Search strategy.

Database	Search Strategy	Search Data
MEDLINE/PubMed	finite element AND (bone AND dental implant*) AND stress AND (anisotropic OR orthotopic OR isotropic) NOT (review)	1 July 2020
Science Direct	finite element AND (bone AND dental implant) AND stress AND (anisotropic OR orthotopic OR isotropic) NOT (review)	1 July 2020
LILACS	finite element AND (bone AND dental implant*) AND stress NOT (review)	1 July 2020

**Table 2:** Main characteristics of dental implants.

Authors	Title	Material /Properties (Young’s Modulus [GPa]/Poisson’s Ratio)	Geometric Characteristic (Diameter/Length [mm])	Manufacturer	Connection
Aslam et al. [8]	Effect of Platform Switching on Peri-Implant Bone: A 3D Finite Element Analysis	Titanium/110/0.35	4.5/11	-	-
Dos Santos et al. [9]	Stress distribution in cylindrical and conical implants under rotational micromovement with different boundary conditions and bone properties: 3-D FEA	Titanium/110/0.35	4.1/11	-	-
Tsouknidas et al. [10]	The Influence of Bone Quality on the Biomechanical Behavior of a Tooth-Implant Fixed Partial Denture: A Three-Dimensional Finite Element Analysis	Titanium/110/0.3	-	Biomed 3i	-
Oswal et al. [11]	Influence of three different implant thread designs on stress distribution: A three-dimensional finite element analysis	Titanium/110/0.3	4/12	-	-
Tsouknidas et al. [12]	Influence of Alveolar Bone Loss and Different Alloys on the Biomechanical Behavior of Internal-and External-Connection Implants: A Three-Dimensional Finite Element Analysis	Titanium/110 and 116/0.3	4/13	Biomed 3i	External and internal
Chang et al. [13]	Biomechanical Effect of a Zirconia Dental Implant-Crown System: A Three-Dimensional Finite Element Analysis	Zirconia/110/0.35	4.1/-	Biomed 3i	-
Lee et al. [14]	Three-dimensional numerical simulation of stress induced by different lengths of osseointegrated implants in the anterior maxilla	Titanium/115/0.35	4.0/(8.5, 10.0, 11.5, 13.0, 15.0)	Nobel Biocare	External
Chun et al. [15]	Influence of Implant Abutment Type on Stress Distribution in Bone Under Various Loading Conditions Using Finite Element Analysis	Titanium/114/0.37	4.3/11.5	Warantec	1-body; internal hex; external hex
Pirmoradian et al. [16]	Finite element analysis and experimental evaluation on stress distribution and sensitivity of dental implants to assess optimum length and thread pitch	Titanium/110/0.35	4.1/8.5; 10; 11.5; 13	-	-
Tian et al. [17]	Angled abutments result in increased or decreased stress on surrounding bone of single-unit dental implants: A finite element analysis	Titanium/110/0.3	4.1/10	-	-
Wu et al. [18]	Biomechanical evaluation of one-piece and two-piece small-diameter dental implants: In-vitro experimental and three-dimensional finite element analyses	Titanium/104/0.3	-	-	External
Liu et al. [19]	The effect of platform switching on stress distribution in implants and periimplant bone studied by nonlinear finite element analysis	Titanium/110/0.33	-	Ankylos and Anthogyr	Internal
Koca et al. [20]	Three-dimensional finite-element analysis of functional stresses in different bone locations produced by implants placed in the maxillary posterior region of the sinus floor	Titanium/110/0.35	4.1/10	ITI	-
Sevimay et al. [21]	Three-dimensional finite element analysis of the effect of different bone quality on stress distribution in an implant-supported crown	Titanium/110/0.35	4.1/10	ITI	-
de Cos Juez et al. [22]	Non-linear numerical analysis of a double-threaded titanium alloy dental implant by FEM	Titanium/110/0.33	-	-	-
Ormianer et al. [23]	Implant-supported first molar restorations: correlation of finite element analysis with clinical outcomes	Titanium/110/0.34	3.7, 4.7, and 6.0/-	-	-
Canullo et al. [24]	The influence of platform switching on the biomechanical aspects of the implant-abutment system. A three-dimensional finite element study	-	3.8 and 5.5/-	-	-

**Table 3:** Finite elements parameters.

Authors	Mesh (Nodes/Elements)	Mesh Element	Software	Bone Type Model (Density)	Cortical Bone Young’s Modulus (GPa)/Poisson’s Ratio	Cancellous Bone Young’s Modulus (Gpa)/Poisson’s Ratio	Loading Conditions	Boundary Conditions	Convergence Criterion
Aslam et al. [8]	76,150/44,208	-	ANSYS Workbench 16	Anisotropic (-)	E_x_: 12.6/0.3 and 0.253 E_y_: 12.6/0.253 and 0.3 E_z_: 19.4/0.39 and 0.39	E_x_: 1.15/0.055 and 0.01 E_y_: 0.21/ 0.322 and 0.01 E_z_: 1.15/0.055 and 0.322	Vertical: 200 to 800 N. Oblique: 50 to 150 N	100% osseointegration. Assembly: constrained in the *x*, *y*, and *z* planes.	-
Dos Santos et al. [9]	75,463/42,740	Tetrahedral with 10 nodes	ANSYS Workbench 11	Isotropic and anisotropic (D2)	E_x_ = 12.6/0.3 E_y_ = 12.6/0.3 E_z_ = 19.4/0.253	E_x_ = 1.148/0.055 Ey = 0.21/0.01 E_z_ = 1.148/0.32	-	100% osseointegration. Border of the models: constrained in all directions.	6%
Tsouknidas et al. [10]	-/704,068	Tetrahedral	ANSYS	Isotropic (-)	13.7/0.33	1.37/0.3	Axial: 200 N in premolar and 230 in molar	100% osseointegration. Bottom surface fixed.	-
Oswal et al. [11]	14,805/72,545	-	ANSYS	Isotropic (-)	13.7/0.30	1.370/0.30	Vertical: 100 N	100% osseointegration. Mandible fixed.	-
Tsouknidas et al. [12]	-	-	ANSYS 15	Isotropic (-)	13.7/0.33	1.37/0.3	200 N/50°	-	-
Chang et al. [13]	47,408 /194,978	Hexahedral	ANSYS 11	Anisotropic (-)	E_y_ = 12.5 E_x_ = 17.9 E_z_ = 26.6 ν_yx_ = 0.18 ν_yz_ = 0.31 ν_xz_ = 0.28	E_y_ = 0.021 E_x_ = 1.148 E_z_ = 1.148 ν_yx_ = 0.055 ν_yz_ = 0.055 ν_xz_ = 0.322	Vertical: 200 N Horizontal: 40 N	100% osteointegration. Symmetric boundary conditions. Mesial surface: constrained in all directions.	-
Lee et al. [14]	-/182,921	-	AMMEDYSA version 2009	Isotropic (D3)	13.7/0.3	1.37/0.3	176 N/120°	Mesial and distal surfaces: fixed in all dimension.	-
Chun et al. [15]	-	Eight nodes	-	Isotropic (-)	14/0.3	1.5/0.3	100 N/15°,30°,60°	Nonlinear contact friction. Outer surface of bone fixed.	-
Pirmoradian et al. [16]	-/153,048	10-node quadratic tetrahedron	ABAQUS (6.14.2)	Isotropic (-)	13.7/0.3	1.37/0.3	180 N/45°	100% osseointegration.	-
Tian et al. [17]	116,428/75,182	SOLID 187	ANSYS 9.0	Isotropic (-)	13.7/0.3	1.37/0.3	100 N	Good osseointegration. The lower surface, the medial and distal planes: completely constrained.	-
Wu et al. [18]	-	SOLID 187	ANSYS Workbench 10.0	Isotropic (-)	16.7/0.3	0.759/0.3	190 N/30°	Contact coefficient abutment-implant: 0.323. Contact coefficient implant- cortical bone: 0.4. Contact cortical-cancellous: 0.8. The mesial and distal surfaces: constrained.	-
Liu et al. [19]	38,744/187,569	-	-	Isotropic (-)	13.4/0.3	1.37/0.3	Vertical: 50, 100 or 150 N Horizontal: 50 and 100 N	-	-
Koca et al. [20]	-	-	Pro/Engineer 2000i	Isotropic (D3)	13.4/0.3	1.37/0.3	300 N	*x*-axis for each design: fixed.	-
Sevimay et al. [21]	32,083/180,884	-	Pro/Engineer 2000i	Isotropic (D1, D2, D3, D4)	13.7/0.3	D1 to D3: 1.37/0.3 D4: 1.1/0.3	300 N	*x*-axis for each design: fixed.	-
de Cos Juez et al. [22]	109,696/295,700	SOLID187	-	Anisotropy (-)	E_1_ = 12.5 E_2_ = 17.9 E_3_ = 26.6 ν_12_ = 0.18 ν_13_ = 0.31 ν_23_ = 0.28	E_1_ = 0.21 E_2_ = 1.148 E_3_ = 1.148 ν_12_ = 0.055 ν_13_ = 0.055 ν_23_ = 0.322	Vertical: 150 N Horizontal: 15 N	The friction between implant and cancellous bone interface was considered to be 0.72.	<0.5%.
Ormianer et al. [23]	-	-	ANSYS Workbench 11	Isotropic (-)	15/-	1.5/-	222 N/30°	Bone implant contact between 8 and 100%.	-
Canullo et al. [24]	100,000/60,000	-	-	Isotropic (-)	15/0.35	1.5/0.3	Vertical: 130 N Horizontal: 90 N	-	-

**Table 4:** Stress on bone and dental implant.

Author	Stress in Dental Implant [MPa]/Criterion	Stress in Bone [MPa]/Criterion
Aslam et al. [8]	Axial load: 178.75/- Oblique: 176.15/ von Mises	Axial load: 300; Oblique: 234/ von Mises
Dos Santos et al. [9]	Isotropic bone: 879.96/- Anisotropic bone 1122.70/-	Isotropic bone: 1076.50; Anisotropic bone: 1433.20/maximum principal stress
Tsouknidas et al. [10]	702/-	42/von Mises
Oswal et al. [11]	21.83/-	Cortical: 3.8909 Cancellous: 1.016/-
Tsouknidas et al. [12]	400–1250/-	5.68–1284/-
Chang et al. [13]	144.69/von Mises	105.52/von Mises
Lee et al. [14]	55.1–59.6/-	4.9–6.9/-
Chun et al. [15]	240–710/-	10–35/von Mises
Pirmoradian et al. [16]	278/von Mises	89.6–93.17/maximum stress
Tian et al. [17]	55/von Mises	55/von Mises
Wu et al. [18]	180/von Mises	90/von Mises
Liu et al. [19]	330/von Mises	8/von Mises
Koca et al. [20]	155/von Mises	50/von Mises
Sevimay et al. [21]	532/-	D3 and D4: 163 and 180/von Mises D1 and D2: 150 and 152/von Mises
de Cos Juez et al. [22]	17.65/von Mises	5.6/von Mises
Ormianer et al. [23]	13–41/-	11–37/-
Canullo et al. [24]	0.064–190/-	0.067 and 52/maximum stress
